# Case of Triplets

**Published:** 1840

**Authors:** 


### CASE OF TRIPLETS.

William C. Lawrence, M. D., of Cincinnati, has sent us a memorandum concerning a case of *triplets*, from which we condense and record the following facts: The patient was a mulatto, who, for two weeks before delivery, was annoyed with spurious labour pains. At 8 P. M., Dr. L. could discover no dilation of the tincæ, and administered opium. Six hours afterwards he was called, and found the first child just delivered and the cord cut. The next presented with its feet and was delivered forthwith. The third presented one foot and one knee, and was delivered without difficulty, but was asphyxiated. By artificial respiration it was restored, and up to the date of the communication, two months, the whole were alive and growing well, under the double supply of the breast and the spoon. The children, two males and one female, were nearly of the same size, and weighed in the aggregate, 17 lbs.

The three placentæ with their membranes, were distinct from each other, and came away without difficulty; but the hemorrhage, as might be expected, was considerable. D.

